# Characterization of Toll-like receptors in primary lung epithelial cells: strong impact of the TLR3 ligand poly(I:C) on the regulation of Toll-like receptors, adaptor proteins and inflammatory response

**DOI:** 10.1186/1476-9255-2-16

**Published:** 2005-11-29

**Authors:** Mirko Ritter, Detlev Mennerich, Andreas Weith, Peter Seither

**Affiliations:** 1 Department of Pulmonary Research, Boehringer-Ingelheim Pharma GmbH & Co. KG, Birkendorfer Straβe, 88937 Biberach a.d. Riss, Germany

## Abstract

**Background:**

Bacterial and viral exacerbations play a crucial role in a variety of lung diseases including COPD or asthma. Since the lung epithelium is a major source of various inflammatory mediators that affect the immune response, we analyzed the inflammatory reaction of primary lung epithelial cells to different microbial molecules that are recognized by Toll-like receptors (TLR).

**Methods:**

The effects of TLR ligands on primary small airway epithelial cells were analyzed in detail with respect to cytokine, chemokine and matrix metalloproteinase secretion. In addition, the regulation of the expression of TLRs and their adaptor proteins in small airway epithelial cells was investigated.

**Results:**

Our data demonstrate that poly(I:C), a synthetic analog of viral dsRNA, mediated the strongest proinflammatory effects among the tested ligands, including an increased secretion of IL-6, IL-8, TNF-α, GM-CSF, GRO-α, TARC, MCP-1, MIP-3α, RANTES, IFN-β, IP-10 and ITAC as well as an increased release of MMP-1, MMP-8, MMP-9, MMP-10 and MMP-13. Furthermore, our data show that poly(I:C) as well as type-1 and type-2 cytokines have a pronounced effect on the expression of TLRs and molecules involved in TLR signaling in small airway epithelial cells. Poly(I:C) induced an elevated expression of TLR1, TLR2 and TLR3 and increased the gene expression of the general TLR adaptor MyD88 and IRAK-2. Simultaneously, poly(I:C) decreased the expression of TLR5, TLR6 and TOLLIP.

**Conclusion:**

Poly(I:C), an analog of viral dsRNA and a TLR3 ligand, triggers a strong inflammatory response in small airway epithelial cells that is likely to contribute to viral exacerbations of pulmonary diseases like asthma or COPD. The pronounced effects of poly(I:C) on the expression of Toll-like receptors and molecules involved in TLR signaling is assumed to influence the immune response of the lung epithelium to viral and bacterial infections. Likewise, the regulation of TLR expression by type-1 and type-2 cytokines is important considering the impact of exogenous and endogenous TLR ligands on Th1 or Th2 driven pulmonary inflammations like COPD or asthma, respectively.

## Background

In addition to its barrier function, the airway epithelium plays an important role for the immune response in the lung [1]. Thus, the lung epithelium is a major source of cytokine, chemokines and other inflammatory mediators that affect the adaptive and innate immune response and therefore modulate inflammatory diseases like COPD or asthma [2]. The innate immune functions of the lung epithelium are critical for the local host defense providing protection of the airways and lung parenchyma from microbial colonization and infections. Besides the induction of acute or chronic pulmonary inflammation, increased bacterial load and viral infections of the lung lead to severe exacerbations of diseases like COPD [3,4] or asthma [5] most probably due to a biased release of inflammatory mediators.

The family of Toll-like receptors (TLR) plays a key role in pathogen recognition and induction and regulation of the innate and adaptive immune response. Airway epithelial cells express TLRs and activation of TLRs on epithelial cells has been shown to induce the production of several cytokines, chemokines and antimicrobial peptides [6-8]. The importance of TLRs for the host defense in the lung has been demonstrated by the increased susceptibility of TLR knockout mice towards viral or bacterial infections. For example, TLR2 deficient mice have been shown to be highly susceptible to infection by *Staphylococcus aureus*, *Borrelia burgdorferi *and *Streptococcus pneumonia *[9,10]. In addition to their prominent role in host response and innate immunity, TLRs play an important role for the adaptive immune system and the regulation of a Th1/Th2 balance [11]. This is thought to have a strong impact for Th2 biased allergic disease like asthma. Whereas the TLR1/TLR2 specific agonist Pam_3_CSK_4 _and high levels of the TLR4 ligand *E. coli *LPS have beneficial effects in asthma animal models most probably due to re-equilibrium of the cytokine pattern and the induction of a Th1 response [12-14], low dose of LPS and the TLR2 ligand peptidoglycan bias the immune response toward a Th2 phenotype and lead to aggravation of experimental asthma [12,15]. In Th1 associated inflammatory disease like COPD, activation of TLRs either by exogenous or endogenous ligands is likely to result in disease exacerbations due to a biased proinflammatory response.

In the current study, we aimed to get a better understanding of the role of TLRs in airway epithelial cells and the consequences for pulmonary inflammatory diseases. Therefore, we performed a detailed analysis of cytokine and chemokine secretion by primary small-airway epithelial cells (SAEC) induced by the activation with different TLR ligands. We also analyzed the matrix metalloproteinase (MMP) release of the stimulated SAEC. Furthermore, the regulation of the expression of TLRs and their adaptor proteins in primary airway epithelial cells (SAEC) stimulated with TLR ligands or Th1 and Th2 cytokines was investigated. We could demonstrate that among the different TLR ligands evaluated poly(I:C), an analog of viral dsRNA and a ligand for TLR3, is the most potent proinflammatory stimulus for lung epithelial cells regarding the secretion of cytokines, chemokines and MMPs. Poly(I:C) induced an increased expression of its receptor TLR3 and has pronounced effects on the expression of other TLRs and proteins involved in TLR signaling in SAEC. In addition, we demonstrate that the expression of TLRs and their signaling proteins in SAEC is strongly regulated by type-1 and type-2 cytokines. These findings are thought to have a major effect on the impact of bacterial and viral infections in type-1 or type-2 biased pulmonary inflammations like COPD or asthma, respectively. In summary, our results provide further insight in the role of TLRs in the immune response of the lung epithelium to viral and bacterial infections and their contribution to virus and bacteria induced exacerbations of pulmonary diseases.

## Methods

### Antibodies

Polyclonal antibodies to TRIF and TIRAP and mAb to TOLLIP were obtained from Alexis (Lausen, Switzerland). Polyclonal antibodies to IRAK-2 and IRAK-3 were purchased from Chemicon (Temecula, CA). Polyclonal antibodies to IRAK-1 and IRAK-4 were from Active Motif (Rixensart, Belgium) or Upstate (Waltham, MA), respectively. The polyclonal anti-MyD88 antibody was obtained from Santa-Cruz Biotechnology (Santa-Cruz, CA). The anti-TLR1 (GD2.F4) mAb, anti-TLR2 mAb (TL2.3), anti-TLR3 mAb (TLR3.7) used for immunofluorescence was purchased from Alexis. The anti-TLR5 mAb and anti-TLR6 mAb (86B1153) were obtained from Imgenex (San Diego, CA) and Kamiya (Seattle, WA), respectively. The neutralizing goat polyclonal anti-IFN-β antibody was from R&D Systems (Wiesbaden, Germany).

### Cell Culture

Primary SAEC (normal human small airway epithelial cells) as well as all the basal media and growth supplements were obtained from Clonetics (San Diego, CA). Cells were cultivated according to the instructions of the manufacturer on plastic dishes or flasks (BD Bioscience, Heidelberg, Germany). Passage number was kept to less than four passages from original stocks. SAEC cells were maintained in small airway epithelial cell basal medium (SAGM) supplemented with 52 μg/ml bovine pituitary extract, 0.5 ng/ml human recombinant EGF, 0.5 μg/ml hydrocortisone, 0.5 μg/ml epinephrine, 10 μg/ml transferrin, 5 μg/ml insulin, 0.1 ng/ml retinoic acid (RA), 6.5 ng/ml triiodothyronine, 50 μg/ml Gentamicin/Amphotericin-B (GA-1000) and 50 μg/ml fatty acid-free bovine serum albumin (BSA). 24 h before stimulation of the cells medium was replaced by basal medium only supplemented with GA-1000, RA and BSA. SAECs of three different donors were used for the study.

### Stimulation of small-airway epithelial cells (SAEC)

All cytokines were obtained from R&D Systems (Wiesbaden, Germany). The following cytokine concentrations were used for stimulation of SAECs: 50 ng/ml TNF-α, 10 ng/ml IL-1β, 10 ng/ml IL-4, 20 ng/ml IL-13 or 20 ng/ml IFN-γ.

To evaluate the effects of TLR activation, SAEC were stimulated with 5 μg/ml *E. coli *0111:B4 LPS (ultrapure), 10 μg/ml *S. aureus *peptidoglycan (PGN), 10 μg/ml *S. cerevisiae *zymosan, 200 ng/ml MALP-2 (macrophage activating lipopeptide-2), 200 ng/ml Pam_3_CSK_4 _(palmitoyl-3-cysteine-serine-lysine-4) (all obtained from Invivogen, (San Diego, CA), 10 μg/ml poly(I:C) (SIGMA, Munich, Germany) or 20 ng/ml *S. muenchen *flagellin (Calbiochem, Darmstadt, Germany).

24 h before stimulation of the cells, medium was replaced by basal medium only supplemented with GA-1000, retinoic acid and BSA. Subsequently, cells were incubated for 6 h or 24 h with the indicated stimuli in basal SAGM medium supplemented with GA-1000, retinoic acid and BSA.

For blocking experiments, cells were pre-incubated with a functional blocking anti-TLR3 mAb (clone TLR3.7; 20 μg/ml) [16], an goat anti-IFN-β antibody (1 – 5 μg/ml), an isotype control IgG_1 _(20 μg/ml) or a goat IgG (1 – 5 μg/ml) for 1 h and subsequently stimulated with 5 μg/ml poly(I:C) for 6 h. IP-10 and IFN-β secretion were measured using an IP-10 or IFN-β ELISA (R&D Systems), respectively.

### RNA preparation

RNA extraction from cells was carried out according to the manufacturer's instructions using the RNeasy Mini Kit (Qiagen, Hilden, Germany). Purity and integrity of the extracted RNA was assessed on the Agilent 2100 bioanalyzer with the RNA 6000 Nano LabChip reagent set (Agilent Technologies, Palo Alto, CA).

### Real-time quantitative RT-PCR

Primers and TaqMan probes for human TLR1 – TLR5 and TRAM (table 1) were designed using the PrimerExpress Software 2.0 (Applied Biosystems, Darmstadt, Germany). The probes used for detection in real-time PCR were labeled with 6-carboxyfluorescein (FAM) at their 5'-terminal and were quenched with 6-carboxytetramethylrhodamine (TAMRA) on their 3'-terminal. Primers and TaqMan probes for human β-actin, TLR6 – TLR10, IRAK-1, IRAK-2, IRAK-3, IRAK-4, TRIF, TIRAP, TOLLIP, I-TAC, IP-10 and MyD88 were obtained from Applied Biosystems (Assay-On-Demand). GAPDH mRNA levels were measured using a JOE labeled human GAPDH TaqMan PDAR endogenous control reagent kit (Applied Biosystems).

**Table 1:** Primers and probes used for quantitative real-time RT-PCR

**Oligonucleotide**	**Sequence**
TLR1 (FP)	5'-CCCATTCCGCAGTACTCCATT-3'
TLR1 (RP)	5'-TTTCCTTGGGCCATTCCA-3'
TLR1 (TP)	5'-CAGTTATCACAAGCTCAAAAGTCTCATGGCCA-3'
TLR2 (FP)	5'-TGTGAAGAGTGAGTGGTGCAAGT-3'
TLR2 (RP)	5'-ATGGCAGCATCATTGTTCTCAT-3'
TLR2 (TP)	5'-TGAACTGGACTTCTCCCATTTCCGTCTTTT-3'
TLR3 (FP)	5'-CCTGGTTTGTTAATTGGATTAACGA-3'
TLR3 (RP)	5'-GAGGTGGAGTGTTGCAAAGGTAGT-3'
TLR3 (TP)	5'-CCCATACCAACATCCCTGAGCTGTCAA-3'
TLR4 (FP)	5'-AGCTCTGCCTTCACTACAGAGACTT-3'
TLR4 (RP)	5'-GCTTTTATGGAAACCTTCATGGA-3'
TLR4 (TP)	5'-CCCGGTGTGGCCATTGCTGC-3'
TLR5 (FP)	5'-GCACTTTTATCAATTGGCTTAATCAC-3'
TLR5 (RP)	5'-AACGAGTCAGGGTACACACAATATATG-3'
TLR5 (TP)	5'-CAATGTCACTATAGCTGGGCCTCCTGCAG-3'
TRAM (FP)	5'-CAGTGCTCTTACCCAGATGGA-3'
TRAM (RP)	5'-TCTGATAATCGATGACAGACTTCA-3'
TRAM (TP)	5'-CTGCCTGTGTTTCAATTCACGAAGCT-3'

FP: forward primer; RP: reverse primer; TP: FAM and TAMRA-labeled TaqMan probe

TaqMan PCR assays were performed on an ABI Prism 9600 Sequence Detection System (Applied Biosystems) as a one-step RT-PCR using the EZ-RT-PCR Reagent Kit (Applied Biosystems) and 40 ng of RNA. Final Mn^2+ ^concentrations were optimized for each assay and varied between 2.5 mM and 4 mM. Assays were performed in 384-well optical plates and run in duplicates or triplicates. To quantify the results obtained by real-time PCR, we used a calibration curve. Serial dilutions of human lung or spleen RNA were used as standards and were run in parallel to the samples. The Sequence Detector Software SDS 2.0 (Applied Biosystems) was used for data analysis. The results were normalized to endogenous controls (GAPDH or β-actin).

### Immunofluorescence

Immunocytochemistry for TLRs was performed on human primary small airway epithelial cells grown on collagen-I coated chamber slides (BD Bioscience) in completely supplemented SAGM. 24 h before stimulation of the cells medium was replaced by basal medium only supplemented with GA-1000, retinoic acid and BSA. Subsequently, cells were stimulated for 24 h with the indicated compounds. For staining cells were fixed and permeabilized by incubation with BD Cytofix/Cytoperm solution (BD Bioscience). The anti-TLR1 (GD2.F4) mAb, anti-TLR2 mAb (TL2.3), anti-TLR3 mAb (TLR3.7) and anti TLR5 (19D759.2) mAbs were used at a concentration of 2 – 5 μg/ml. Antibody dilutions and washing was performed in BD Perm/Wash solution (BD Bioscience). To rule out non-specific staining, a matching isotype negative control was used instead of the TLR specific antibody. Binding of the primary antibody was detected using an Alexa488-labeled anti-mouse antibody (Molecular Probes, Eugene, OR). Nuclei were stained with propidium iodide.

### Measurement of cytokine, chemokine and MMP secretion

To analyze the secretion of chemokines and cytokines by SAEC, cells were stimulated for 24 h with different ligands for TLRs as described above. Cell supernatants were cleared by centrifugation, supplemented with a protease inhibitor mix (Complete™, Roche) and stored at -80°C. Cytokines and chemokines were detected using a cytokine array (RayBio^® ^Human Cytokine Antibody Array III, RayBiotech, Norcross, GA) following the manufacturer's instructions. A complete list of cytokine antibodies present on the array is available under .

For quantification of chemokines, MMPs multiplex assays were performed in duplicates using three different dilutions of the cell supernatants. Assays were performed according to the manufacturer's instructions (SearchLight™ Array, Sample Testing Service, Pierce, Woburn, MA).

### Western blotting

Western blotting was performed according to standard procedures. Briefly, proteins were separated by SDS-PAGE under reducing conditions and blotted on PVDF membranes. Membranes were blocked in PBS/5% milk powder/0.1% Tween-20. Primary and secondary antibodies were diluted in PBS/5% milk powder/0.1% Tween-20. Antibody binding was detected using a HRP labeled secondary antibody and SuperSignal^® ^West Pico chemiluminescence substrate (Pierce).

## Results

### TLR triggered secretion of cytokines and chemokines by SAEC

Since the lung epithelium is a major source of chemokine and cytokine secretion in various viral and bacterial pulmonary infections [1,17], we analyzed which types of cytokines and chemokines are released from the small airway epithelial cells (SAEC) in response to activation by different TLR ligands.

**Figure 1:**
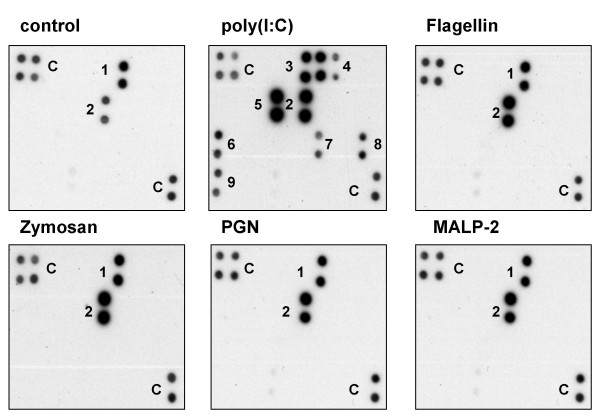
**Cytokine and chemokine secretion by stimulated primary small airway epithelial cells (SAEC)**. SAEC were stimulated for 24 h with different TLR ligands including poly(I:C), flagellin, zymosan, peptidoglycan (PGN) and macrophage activating lipopetide-2 (MALP-2) and cell supernatants were analyzed using a cytokine antibody array. Cytokines and chemokines were detected by chemiluminescence. Results were compared to untreated controls. The figure shows representative results of three independent experiments. (C) internal positive control, (1) GRO, (2) IL-8, (3) GM-CSF, (4) GRO-α, (5) IL-6, (6) MCP-1, (7) RANTES, (8) TARC, (9) TNF-α.

**Figure 2:**
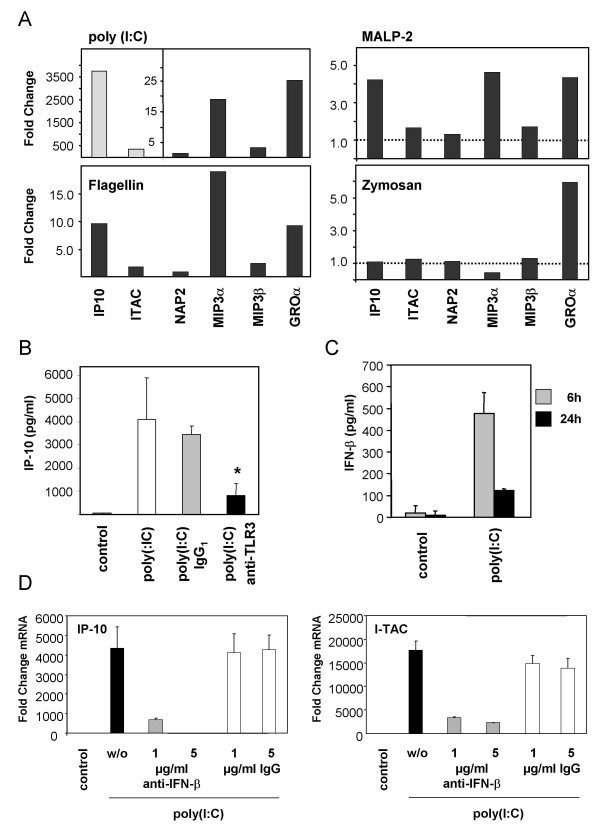
**Chemokine secretion by stimulated primary small airway epithelial cells (SAEC)**. **(A) **SAEC were stimulated for 24 h with different TLR ligands including poly(I:C), flagellin, zymosan and macrophage activating lipopetide-2 (MALP-2) and cell supernatants were analyzed using multiplex ELISAs. Results are shown as fold changes relative to untreated controls. The figure shows representative results of three independent experiments. Stimulation of the cells with LPS or PGN had no significant effect on the secretion of the indicated chemokines (data not shown). **(B) **Inhibition of poly(I:C) induced IP-10 secretion by a monoclonal, functional blocking anti-TLR3 antibody. SAEC were stimulated in the presence of an anti-TLR3 antibody or an IgG_1 _isotype control with 5 μg/ml poly(I:C) for 6 h and IP-10 secretion was analyzed in triplicates using an IP-10 ELISA. Results were compared to untreated controls and poly(I:C) stimulated cells in the absence of an antibody. **(C) **IFN-β secretion of SAEC after stimulation with poly(I:C). SAEC were stimulated with 5 μg/ml poly(I:C) for 6 h or 24 h and IFN-β secretion was analyzed in triplicates using an IFN-β ELISA. Results were compared to untreated controls. **(D) **Inhibition of poly(I:C) induced IP-10 and I-TAC expression by a goat polyclonal functional blocking anti-IFN-β antibody. SAEC were stimulated in the presence of a goat anti-IFN-β antibody (1 or 5 μg/ml; gray bars) or a goat IgG control (1 or 5 μg/ml; white bars) with 5 μg/ml poly(I:C) for 6 h. IP-10 or I-TAC expression was analyzed by real-time RT-PCR. Expression data were normalized using β-actin as endogenous control and are shown as fold changes relative to untreated controls. Results were compared to poly(I:C) stimulated cells in the absence of an antibody (black bars).

Using a real-time RT-PCR approach we could demonstrate that human SAEC constitutively express mRNA of TLRs 1–6, whereas expression of TLRs 7–10 was not detected (data not shown). Therefore, SAECs were stimulated for 24 h with different ligands of TLR1/TLR2 or TLR2/TLR6 heterodimers (PGN, zymosan, Pam_3_CSK_4_, MALP-2), TLR3 (poly(I:C)), TLR4 (*E. coli *LPS) or TLR5 (flagellin). After stimulation, cytokine and chemokine levels in the cell culture supernatants were analyzed using a cytokine array (Fig. 1) and multiplex ELISA systems (Fig. 2A).

As shown in Fig. 1, stimulation of SAEC with the TLR3 ligand poly(I:C) resulted in the most pronounced inflammatory response regarding the secretion of cytokines and chemokines including an increased release of IL-6, IL-8, TNF-α, GM-CSF, MCP-1, RANTES, TARC and GRO-α. In contrast to poly(I:C), ligands for TLR2 (MALP-2, PGN, zymosan) or TLR5 (flagellin) induced only an increased secretion of IL-8 compared to the untreated control cells. In all experiments SAEC showed no response to stimulation with LPS regarding the secretion of cytokines or chemokines (data not shown).

To screen an additional panel of chemokines multiplex ELISAs were performed (Fig. 2A). These results supported our initial findings that activation of TLR3 by poly(I:C) leads to a strong proinflammatory response that is characterized by an increased secretion of MIP-3α, MIP-3β and GRO-α and by a very strong induction of the IFN-inducible chemokines IP-10 (CXCL10) and ITAC (CXCL11) (Fig. 2A). In accordance with the induction of IFN-inducible chemokines, we detected an elevated secretion of IFN-β after 6 h of poly(I:C) stimulation (Fig. 2C). The strong induction of IP-10 secretion could be inhibited by a functional blocking anti-TLR3 antibody demonstrating the involvement of cell-surface expressed TLR3 in the response of SAEC to poly(I:C) (Fig. 2B). Likewise, the increased IP-10 and I-TAC expression in poly(I:C) stimulated cells were significantly inhibited by a neutralizing anti-IFN-β antibody indicating an IFN-β dependent mechanism of IP-10 and I-TAC induction by poly(I:C) (Fig. 2D).

In comparison to poly(I:C), stimulation of SAEC with the TLR5 ligand flagellin resulted in a less pronounced secretion of the IFN-inducible chemokine IP-10, but induced the secretion of similar levels of MIP3α, MIP3β and GRO-α (Fig. 2A). MALP-2, a specific ligand for TLR2/TLR6 heterodimers, increased the secretion of IP-10, MIP-3α and GRO-α. The TLR2 ligand zymosan induced an elevated secretion of GRO-α (Fig. 2A). Other TLR2 ligands including Pam_3_CSK_4 _and PGN had no significant impact on the secretion of the analyzed chemokines (data not shown). Again SAEC showed no response to stimulation with the TLR4 ligand LPS (data not shown).

### TLR triggered secretion of matrix metalloproteinases by SAEC

**Figure 3:**
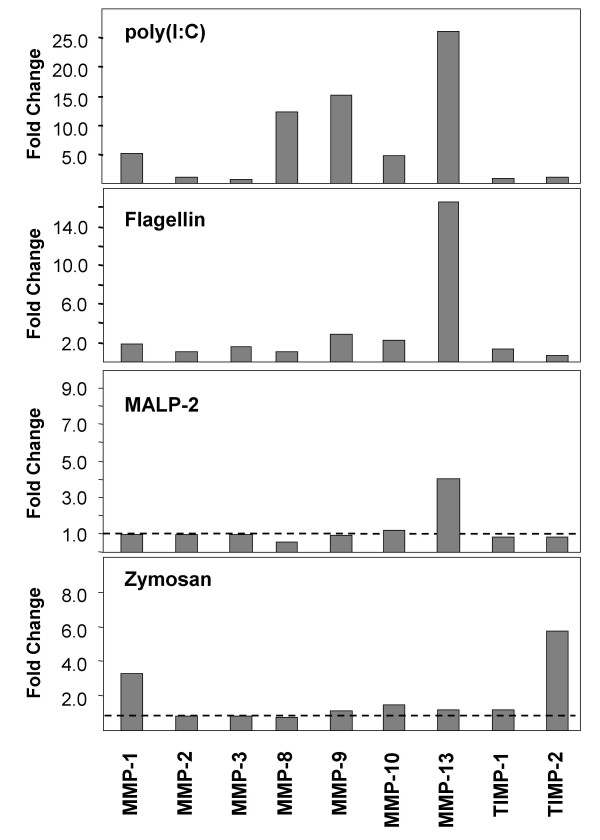
**Matrix metalloproteinase (MMP) and TIMP secretion by stimulated primary small airway epithelial cells (SAEC)**. SAEC were stimulated for 24 h with different TLR ligands including poly(I:C), flagellin, macrophage activating lipopetide-2 (MALP-2) and zymosan and cell supernatants were analyzed using MMP multiplex ELISAs. Results are shown as fold changes relative to untreated controls. The figure shows representative results of three independent experiments.

Since increased levels of matrix metalloproteinases (MMPs) are found in chronically inflamed tissues in diseases like COPD and are thought to contribute to the pathophysiology of the diseases, we analyzed the secretion of MMPs by SAEC and the effect of TLR activation on this process. SAECs were stimulated for 24 h with different ligands for TLRs and MMP concentrations in the cell culture supernatants were analyzed using a multiplex ELISA (Fig. 3).

These results demonstrate that the TLR3 ligand poly(I:C) induced an markedly increased secretion of type-I collagenases MMP-1, MMP-8, MMP-13 as well as an increased release of the type-IV collagenase MMP-9 (Gelatinase B) and the stromelysin MMP-10. Secretion of MMP inhibitors TIMP-1 and TIMP-2 was not elevated. TLR5 activation by flagellin slightly increased the release MMP-1, MMP-9, MMP-10 and induced a strongly elevated secretion of MMP-13 by SAEC. Activation of SAECs with TLR2 ligands had only a minor effect on MMP or TIMP secretion. The TLR2 ligand zymosan induced the release of MMP-1 and the MMP inhibitor TIMP-2. MALP-2, a ligand for TLR2/TLR6 heterodimers, increased the secretion of MMP-13, whereas the TLR1/TLR2 specific ligand Pam_3_CSK_4 _and the TLR4 ligand LPS had no significant effects on the secretion of the analyzed MMPs or TIMPs (data not shown). These data demonstrate that among the tested TLR ligands, poly(I:C) induced the highest MMP secretion by activated lung epithelial cells. This is in accordance with the highly increased cytokine and chemokine secretion induced by poly(I:C) and emphasizes the strong proinflammatory impact of the TLR3 ligand poly(I:C) for SAEC.

### Regulation of TLR expression in SAEC by different ligands for TLRs

**Figure 4:**
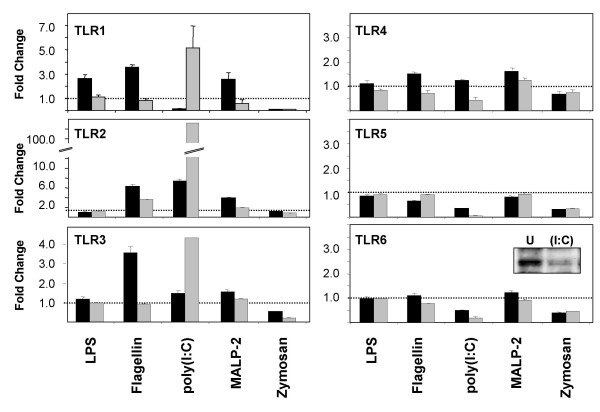
**Toll-like Receptor (TLR) mRNA expression by stimulated primary small-airway-epithelial cells (SAEC)**. SAEC were stimulated for 6 h (black bars) or 24 h (gray bars) with different TLR ligands including LPS, flagellin, poly(I:C), macrophage activating lipopetide-2 (MALP-2) and zymosan. TLR1 – TLR6 mRNA expression was analyzed by quantitative RT-PCR. Results were normalized using β-actin as endogenous control and are shown as fold changes relative to untreated controls. TLR6 protein expression in unstimulated (U) and poly(I:C) stimulated (I:C) SAEC was analyzed by Western blotting and is shown as an insert in the diagram.

**Figure 5:**
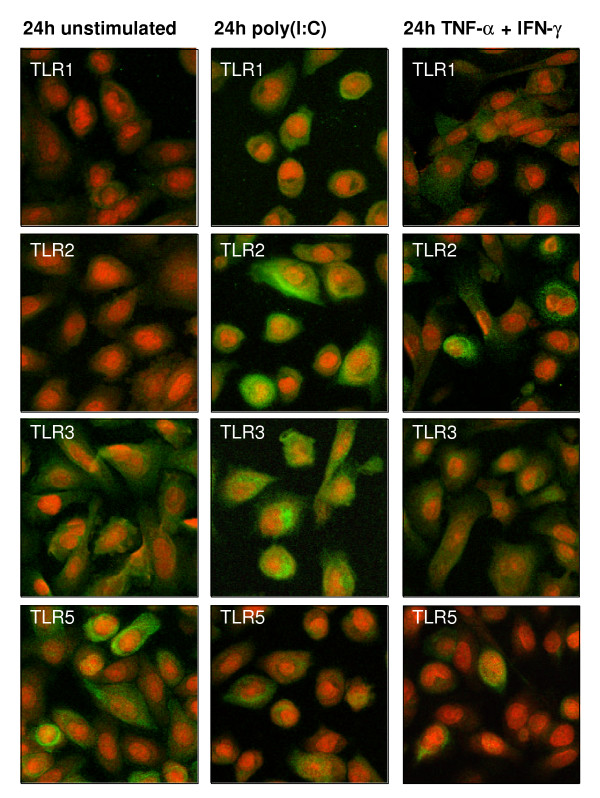
**Toll-like Receptor (TLR) expression in unstimulated and stimulated primary small airway epithelial cells (SAEC)**. SAEC were stimulated for 24 h with poly(I:C) (middle) or with TNF-α in combination with IFN-γ (right). TLR1, TLR2, TLR3 and TLR5 protein expression was analyzed by immunofluorescence of fixed and permeabilized cells (green). Nuclei were stained with propidium iodide (red). Results were compared to unstimulated cells (left).

To analyze whether TLR activation has an effect on the expression of TLRs in SAECs, cells were stimulated for 6 h or 24 h with ligands for TLR2 (PGN, zymosan, Pam_3_CSK_4_, MALP-2), TLR3 (poly(I:C)), TLR4 (*E. coli *LPS) or TLR5 (flagellin) and TLR mRNA and protein expression was measured by quantitative real time RT-PCR and immunofluorescence. As shown in Fig. 4, the TLR3 ligand poly(I:C) had the most potent effects on the mRNA expression of TLRs in SAEC. We detected an increased mRNA expression of TLR3 after poly(I:C) stimulation that was accompanied with an increased TLR3 protein expression (Fig. 5).

TLR1 mRNA expression was down-regulated after 6 h of poly(I:C) stimulation followed by an up-regulation after 24 h of poly(I:C) stimulation (Fig. 4) that was associated with an elevated TLR1 protein expression (Fig. 5). We also observed a very strong up-regulation of TLR2 mRNA and protein expression in poly(I:C) stimulated SAECs (Fig. 4 and 5). In contrast, mRNA and protein expression of TLR6 was strongly decreased by poly(I:C) (Fig. 4). Likewise, we observed a strong down-regulation of TLR5 mRNA (Fig. 4) and protein (Fig. 5) expression by poly(I:C). In contrast to poly(I:C), other TLR ligands had only minor effects on the regulation of TLR expression in SAECs. We detected no induction of TLR7 – TLR10 gene expression by stimulation of SAECs with poly(I:C) or other TLR ligands (data not shown).

### Regulation of the expression of TLR signaling molecules by different ligands for TLRs

**Figure 6:**
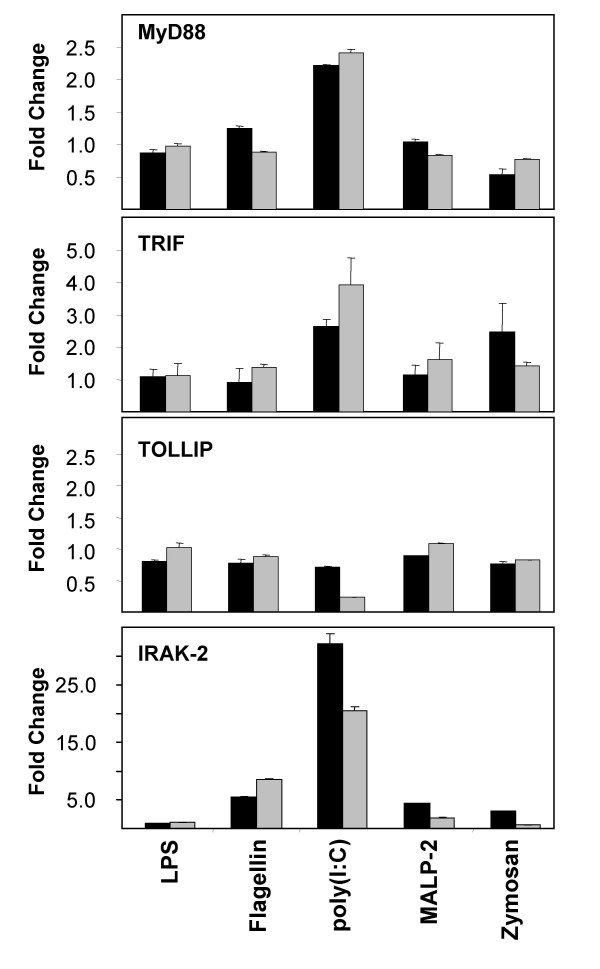
**mRNA expression of genes involved in TLR signaling in stimulated primary small airway epithelial cells (SAEC)**. SAEC were stimulated for 6 h (black bars) or 24 h (gray bars) with different TLR ligands including LPS, flagellin, poly(I:C), macrophage activating lipopetide-2 (MALP-2) and zymosan. MyD88, TRIF, TOLLIP and IRAK-2 mRNA expression was analyzed by quantitative RT-PCR. Results were normalized using β-actin as endogenous control and are shown as fold changes relative to untreated controls. No changes of the gene expression of TRAM, TIRAP, IRAK-1, IRAK-3 and IRAK-4 were observed in the stimulated cells (data not shown).

**Figure 7:**
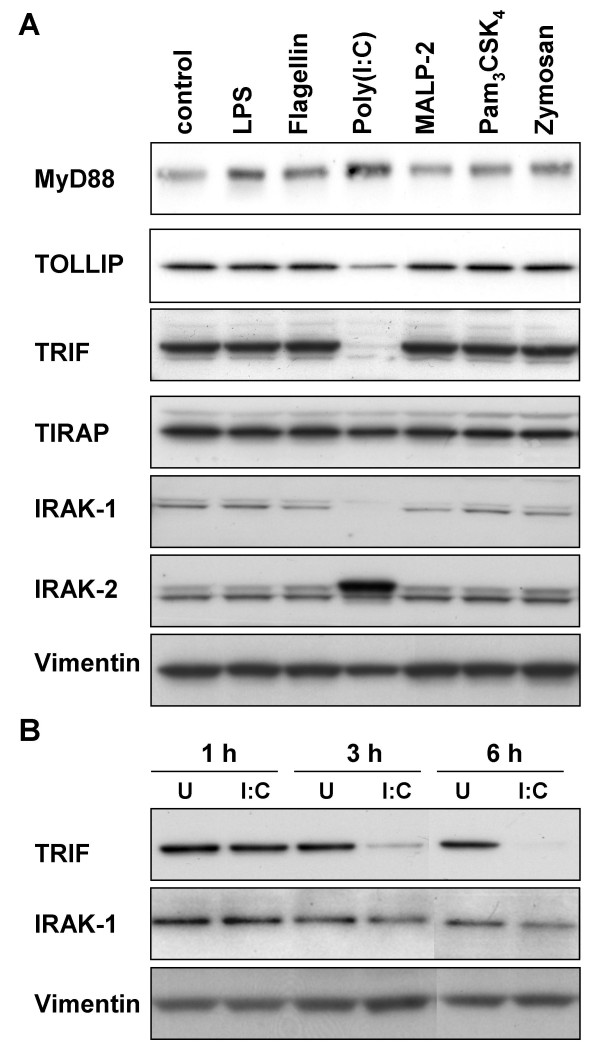
**Expression of proteins involved in TLR signaling in stimulated primary small-airway-epithelial cells (SAEC). (A) **SAEC were untreated (control) or stimulated for 24 h with the indicated TLR ligands. MyD88, TOLLIP, TRIF, TIRAP, IRAK-1 and IRAK-2 protein expression was analyzed by Western blotting. A vimentin loading control is shown below. **(B) **SAEC were stimulated for 1 h, 3 h or 6 h with poly(I:C) (I:C) and TRIF protein levels were analyzed by Western blotting (upper panel). Results were compared to untreated controls (U) and to IRAK-1 protein expression (lower panel).

Signaling by TLRs is initiated by the recruitment of a specific set of adaptor proteins [18]. Since the use of different adaptor proteins provides a mechanism to modulate and specify the response of individual TLRs, we analyzed the regulation of the expression of the known TLR adaptor proteins in small-airway epithelial cells. To examine the impact of different TLR ligands on the expression of the adaptor proteins, cells were stimulated for 6 h or 24 h with different ligands for TLR2 (PGN, zymosan, Pam_3_CSK_4_, MALP-2), TLR3 (poly(I:C)), TLR4 (*E. coli *LPS) or TLR5 (flagellin) and TLR adaptor expression was measured by quantitative real time RT-PCR (Fig. 6) and Western blotting (Fig. 7). As shown in Fig. 6, the TLR3 ligand poly(I:C) had the most potent effects on the mRNA expression of TLR adaptor proteins. Poly(I:C) induced an increased mRNA expression of the common adaptor protein MyD88 and the TLR3 and TLR4 specific adaptor protein TRIF after 24 h of poly(I:C) stimulation. In contrast, the gene expression of TOLLIP, a negative regulator of TLR signaling, was decreased by poly(I:C) (Fig. 6). In accordance with the reduced mRNA expression, TOLLIP protein levels were found to be decreased by poly(I:C) stimulation (Fig. 7A). Surprisingly, Western blot analysis demonstrated that despite the elevated TRIF mRNA expression, TRIF protein levels are strongly decreased after 3 h, 6 h and 24 h of poly(I:C) stimulation indicating that TRIF is cleaved or degraded following TLR3 signaling (Fig 7A and 7B).

Interleukin-1 receptor-associated kinases (IRAKs) are part of the TLR signaling cascade and are involved in the activation of NFκB and the MAP kinase pathway following TLR activation [19]. Due to their most up-stream location in the TLR signaling cascade, we aimed to analyze the regulation of these key signaling proteins in SAECs by TLR ligands. IRAK expression was measured by quantitative real time RT-PCR and Western blotting. As shown in Fig. 6 and Fig. 7A, the TLR3 ligand poly(I:C) had a pronounced effect on the mRNA and protein expression of IRAKs. We found a strongly increased mRNA (Fig. 6) and protein (Fig. 7A) expression IRAK-2 after 24 h of poly(I:C) stimulation, whereas the protein expression of IRAK-1 was strongly decreased by 24 h of poly(I:C) most probably due to an increased IRAK-1 degradation following enhanced TLR signaling (Fig. 7A) since we found no evidence for a transcriptional regulation of IRAK-1 by poly(I:C) (data not shown). IRAK-1 degradation was detectable after 24 h of poly(I:C) stimulation and succeeded TRIF cleavage or degradation (Fig. 7B). This is in agreement with the finding that IRAK-1 activation is down-stream of TRIF in the TLR3 signaling pathway. Poly(I:C) and other TLR ligands had no effect on the mRNA or protein expression of IRAK-3 or IRAK-4 (data not shown).

### Regulation of TLR expression in SAEC by Th1 and Th2 cytokines

**Figure 8:**
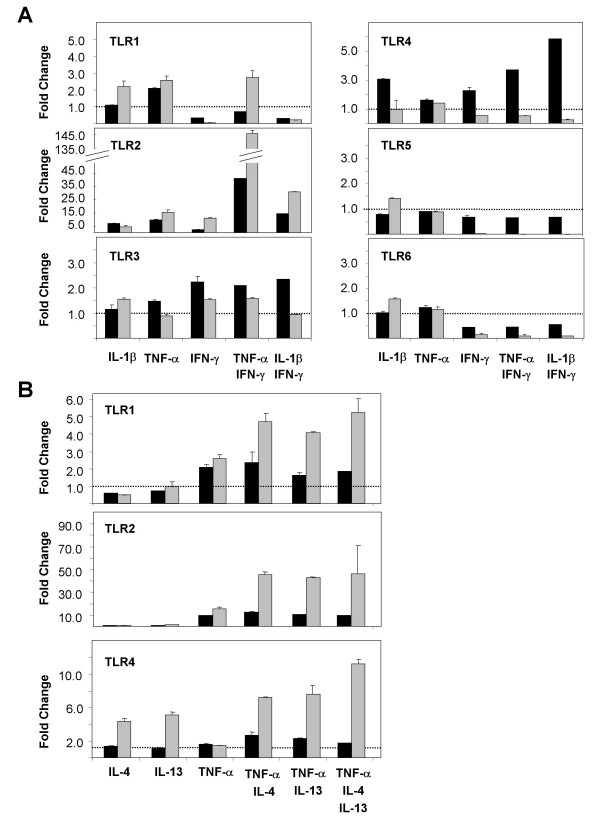
**mRNA expression of Toll-like Receptors (TLR) in stimulated primary small-airway-epithelial cells (SAEC). (A) **SAEC were stimulated for 6 h (black bars) or 24 h (gray bars) with different type-1 cytokines. TLR1 – TLR6 mRNA expression was analyzed by quantitative RT-PCR. Results were normalized using GAPDH as endogenous control and are shown as fold changes relative to untreated controls. **(B) **SAEC were stimulated for 6 h (black bars) or 24 h (gray bars) with different type-2 cytokines. TLR1, TLR2 and TLR4 mRNA expression was analyzed by quantitative RT-PCR. Results were normalized using GAPDH as endogenous control and are shown as fold changes relative to untreated controls. No changes of the gene expression of TLR3, TLR5 and TLR6 were observed by stimulation of SAEC with type-2 cytokines (data not shown).

Since TLR ligands are thought to induce a pronounced type-1 immune response and since the cytokine milieu might affect the TLR expression in SAEC, we investigated the regulation of TLR expression by different type-1 or type-2 cytokines. Therefore, SAEC were stimulated for 6 h or 24 h with cytokines or combinations of cytokines that are associated with a type-1 or type-2 inflammatory response and the expression of TLRs was analyzed by real-time RT-PCR (Fig. 8) and immunofluorescence (Fig. 5). Type-1 cytokines including IL-1β, TNF-α and IFN-γ induced a strong up-regulation of TLR2 mRNA (Fig. 8) and protein expression that was most pronounced by the co-stimulation with TNF-α and IFN-γ (Fig. 5). Likewise, TNF-α in combination with IFN-γ induced an elevated mRNA (Fig. 8A) and protein expression of TLR1 (Fig. 5) and strongly reduced the expression of TLR6 indicating that this cytokine combination favors a signaling through TLR1/TLR2 heterodimers.

Surprisingly, in contrast to the very strong induction of TLR2 mRNA by IFN-γ, both TLR1 and TLR6, which are known to form functional heterodimers with TLR2, were found to be down-regulated by IFN-γ stimulation in the absence of TNF-α (Fig. 8A). Likewise, we observed a strong down-regulation of TLR5 mRNA (Fig. 8A) and protein expression (Fig. 5) after 24 h of TNF-α and IFN-γ stimulation. In contrast to the poly(I:C) mediated up-regulation of TLR3 mRNA and protein expression, we detected no effect of type-1 cytokines on the expression level of TLR3 (Fig. 5 and 8A).

Type-2 cytokines like IL-4 or IL-13 in combination with TNF-α induced in a synergistic manner a strong up-regulation of TLR2 mRNA expression (Fig. 8B). Similar, there is an increase in TLR1 mRNA expression after stimulation with IL-4 or IL-13 in combination with TNF-α, whereas there is no effect on the TLR6 mRNA level (data not shown) indicating that these conditions favor a signaling through TLR1/TLR2 heterodimers. We found no effect on type-2 cytokines on the mRNA expression of TLR3 or TLR5 (data not shown).

In addition to the cytokine milieu, kinetic seems to play an important role in the regulation of TLR4 mRNA expression. Whereas type-1 cytokines induce a rapid up-regulation of TLR4 mRNA levels after 6 h of stimulation that return to a basal level after 24 h of stimulation (Fig. 8A), type-2 cytokines induce a slow increase of TLR4 mRNA levels that is detectable after 24 h of stimulation (Fig. 8B).

### Regulation of TLR signaling proteins in SAEC by Th1 and Th2 cytokines

**Figure 9:**
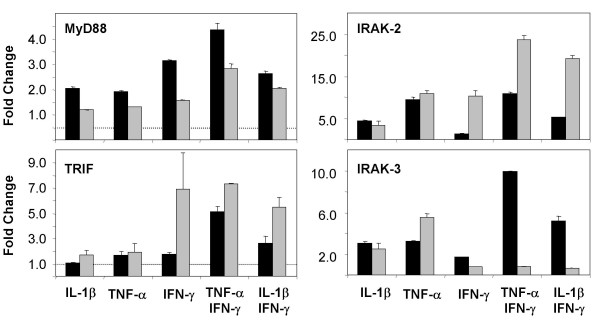
**mRNA expression of genes involved in TLR signaling in stimulated primary small-airway-epithelial cells (SAEC)**. SAEC were stimulated for 6 h (black bars) or 24 h (gray bars) with different cytokines. MyD88, TRIF, IRAK-2 and IRAK-3 mRNA expression was analyzed by quantitative RT-PCR. Results were normalized using GAPDH as endogenous control and are shown as fold changes relative to untreated controls. No changes of the gene expression of TRAM, TIRAP, TOLLIP, IRAK-1 and IRAK-4 were observed in the cytokine stimulated cells (data not shown).

**Figure 10:**
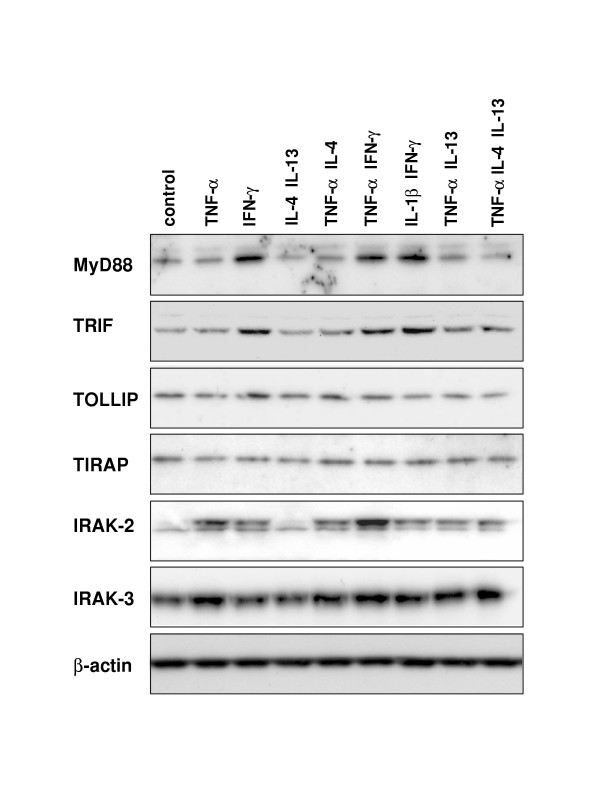
**Expression of proteins involved in TLR signaling in stimulated primary small-airway-epithelial cells (SAEC)**. SAEC were stimulated for 24 h with the indicated cytokines. MyD88, TRIF, TOLLIP, TIRAP, IRAK-2 and IRAK-3 protein expression was analyzed by Western blotting. A β-actin loading control is shown below. No changes of the protein expression of IRAK-1 and IRAK-4 were observed in the stimulated cells (data not shown).

To analyze the regulation of TLR signaling molecules by type-1 or type-2 cytokines, SAEC were stimulated for 6 h or 24 h with cytokines or combinations of cytokines that are associated with a type-1 or type-2 inflammatory response and the expression of TLR signaling molecules was analyzed by real-time RT-PCR (Fig. 9) and Western blotting (Fig. 10). The mRNA expression of MyD88 was found to be rapidly up-regulated after 6 h of stimulation with IFN-γ alone or in combination with IL-1β or TNF-α. Similarly, the mRNA expression of TRIF was induced by the type-1 cytokine IFN-γ alone or in combination with IL-1β or TNF-α (Fig. 9). In contrast to type-1 cytokines, stimulation of the cells with Th2 associated cytokines had no effect on MyD88 or TRIF mRNA levels (data not shown). The mRNA expression data of MyD88 and TRIF correlated very well with the MyD88 and TRIF protein levels as determined by Western blotting (Fig. 10). We found no effect of Th1 or Th2 cytokines on the expression level of the adaptor proteins TRAM, TIRAP and TOLLIP in SAEC.

Likewise, the regulation of IRAKs by TLR ligands by type-1 or type-2 cytokines was analyzed by real-time RT-PCR and Western blotting. As shown in Fig. 9, there is a strong regulation of IRAK-2 and IRAK-3 on the level of mRNA expression. IRAK-2 mRNA expression is highly increased following stimulation of SAEC with type-1 cytokines like IL-1β, TNF-α or IFN-γ. Moreover, there is a synergistic up-regulation of IRAK-2 expression induced by stimulation with IFN-γ together with IL-1β or TNF-α. In contrast to Th1 cytokines, type-2 cytokines like IL-4 or IL-13 had no effect on the IRAK-2 expression and displayed no synergism with TNF-α regarding the up-regulation of IRAK-2 mRNA (data not shown). The mRNA expression data of IRAK-2 closely correlated with the IRAK-2 protein levels as determined by Western blotting (Fig. 10).

Considering the regulation of IRAK-3 in SAECs, we found a slightly increased mRNA expression after stimulation of the cells for 6 h with IL-1β or TNF-α, that was further increased by the simultaneously stimulation with IFN-γ. After 24 h of stimulation with IFN-γ in the presence of IL-1β or TNF-α, IRAK-3 mRNA expression decreased to the basal level. In contrast, stimulation of the cells with IL1β or TNF-α alone resulted in a sustained increased IRAK3 mRNA expression that was also detectable after 24 h of stimulation. Type-2 cytokines like IL-4 or IL-13 only slightly increased IRAK-3 mRNA levels and had no major effect on the TNF-α induced up-regulation of IRAK-3 mRNA (data not shown). The mRNA expression data of IRAK-3 correlated with the IRAK-3 protein levels as determined by Western blotting (Fig. 10). We found no evidence for a regulation of IRAK-1 and IRAK-4 by type-1 or type-2 cytokines in SAEC (data not shown).

## Discussion

Bacterial and viral exacerbations play a crucial role in a variety of lung diseases including COPD or asthma most probably due to a biased release of pro-inflammatory mediators. Since the lung epithelium is a major source of inflammatory molecules [1,2,17], we aimed to analyze which cytokines and chemokines are released from the lung epithelium in response to activation by different microbial molecules that are recognized by Toll-like receptors. To characterize the effects of TLR ligands under well controlled *in vitro *conditions, we have chosen primary small airway epithelial cells (SAEC) as a model to study the inflammatory response of the lung epithelium.

Among the TLR ligands evaluated in this study, poly(I:C), a synthetic analog of viral dsRNA and a well characterized ligand for TLR3 [20], mediated the most potent proinflammatory effects in SAEC. Cytokines and chemokines induced by poly(I:C) included IL-6, IL-8, GM-CSF, TNF-α, MIP-3α, GRO-α, IFN-β and IFN-inducible genes like IP-10, ITAC and RANTES, which is in accordance with the activation of IRF-3 following TLR3 stimulation [21]. Likewise, Guillot et al. reported an elevated secretion of IL-6, IL-8 IFN-β and RANTES in poly(I:C) stimulated BEAS-2B cells (human bronchial epithelial cell line) [8]. In accordance with an elevated IFN-β secretion detectable after 6 h of poly(I:C) stimulation, the expression of the IFN-inducible IP-10 and ITAC could be significantly blocked by an IFN-β neutralizing antibody demonstrating the induction of an IFN-response in SAEC by poly(I:C).

Immunofluorescence staining of permeabilized and non-permeabilized SAEC demonstrated a low cell-surface expression of TLR3 and revealed that most of TLR3 protein is found at an intracellular compartment (data not shown). Nevertheless, the strong induction of IP-10 secretion by poly(I:C) could be markedly inhibited by a functional blocking anti-TLR3 antibody demonstrating a key role of cell-surface expressed TLR3 for the response of SAEC to poly(I:C).

The poly(I:C) induced secretion of this set of chemokines and cytokines is part of a pronounced Th1 response leading to the recruitment and activation of neutrophils, macrophages and Th1 cells. Therefore, this strong proinflammatory type-1 immune response mediated by poly(I:C) is very likely to contribute to viral exacerbations found in type-1 pulmonary diseases like COPD. Surprisingly, poly(I:C) also induced the secretion of TARC, which is a well known chemoattractant of Th2 cells [22]. In addition, IL-8, RANTES and GM-CSF have been shown to be involved in the recruitment and survival of eosinophils [23] and contribute to a Th2 immune response. These findings imply a role for TLR3 stimulation not only for Th1 but also for Th2 mediated pulmonary immune responses like allergic asthma.

Flagellin, a ligand for TLR5, is a compound of bacterial flagellae and has been shown to be strong mediator for pulmonary inflammations [24]. However, flagellin stimulation of SAEC induced only a subset of chemokines or cytokines found to be elevated by poly(I:C) stimulation including IL-6, IL-8, MIP-3α and GRO-α. Interestingly, flagellin also induced IP-10 secretion in SAEC pointing to the induction of IFN-dependent genes following TLR5 activation. In contrast to poly(I:C) and flagellin, ligands for TLR2 were less efficient in stimulating cytokine or chemokine release by SAEC. Among the TLR2 ligands tested, MALP-2, a specific ligand for TLR2/TLR6 heterodimers was the strongest inflammatory stimulus. The precise molecular reasons for the low response of SAEC to TLR2 activation and the observed differences between various TLR2 ligands need further experiments, but might be in part due to the observed low level of TLR2 expression in unstimulated SAEC.

Although we detected a low TLR4 mRNA expression in SAEC, we observed no inflammatory response of the cells to LPS stimulation. This is in accordance with recent findings published by Monick et al. [25] showing that primary airway epithelial cells are unresponsive to endotoxin exposure under normal conditions. Since the airway epithelium – like the intestine epithelium – is in constant contact with multiple pathogen-related antigens like LPS, the hyporesponsiveness of these epithelial cells to LPS stimulation [25,26] is assumed to provide a mechanism to dampen the inflammatory response to the constant LPS exposure and to prevent the chronic inflammation of the tissue.

In addition to an elevated secretion of cytokines and chemokines, poly(I:C) also triggered a markedly increased secretion of matrix metalloproteinase (MMP) by SAECs. Increased levels of MMPs are found in chronically inflamed tissues in diseases like COPD and are thought to contribute to the pathophysiology of the disease by matrix degradation in tissue remodeling and emphysema [27]. Our results demonstrate that activation of TLR3 by poly(I:C) triggered an markedly increased secretion of the type-I collagenases MMP-1, MMP-8, MMP-13 as well as the release of the type-IV collagenase MMP-9 and the stromelysin MMP-10. Likewise, flagellin increased the release MMP-1, MMP-9, MMP-10 and MMP-13. Elevated protein levels of MMP-1, MMP-8 and MMP-9 were found in BALF or lung parenchyma of patients with emphysema [28,29]. Although alveolar macrophages and neutrophils are considered to be the major source of MMPs in the respiratory tract, our data demonstrate that SAEC secrete a variety of MMPs in in response to TLR stimulation and therefore are an additional source for increased proteolytic activity in infected airways that might contribute to lung emphysema.

The results discussed above emphasize the strong inflammatory properties of the TLR3 ligand poly(I:C) regarding the activation of lung epithelial cells that are likely to contribute to virus-induced exacerbations of pulmonary diseases like COPD, asthma or lung fibrosis. To analyze the molecular mechanism that is responsible for the strong inflammatory effects of poly(I:C), we evaluated the expression pattern of TLRs and proteins involved in TLR signaling following poly(I:C) stimulation. These data demonstrated that poly(I:C) induced an elevated expression of TLR3 mRNA and protein expression. In addition, poly(I:C) stimulation up-regulated the mRNA expression of the TLR3 adaptor protein TRIF [30,31], whereas the expression of TOLLIP, a negative regulator of TLR signaling [32], was found to be down-regulated by poly(I:C). This transcriptional regulatory mechanism is assumed to promote TLR3 signaling and to contribute to the strong inflammatory effects induced by poly(I:C). An interesting feature of TLR3 signaling in SAEC is the decrease of TRIF protein levels following poly(I:C) stimulation. The exact mechanism that is responsible for the decreased TRIF protein expression is not known, but might be related to protein cleavage or degradation. Protein degradation of TLR signaling proteins has been demonstrated for IRAK-1 [33], IRAK-4 [34] and MyD88 [35]. Interestingly, cleavage of TRIF by the viral protease NS3/4A was recently described by Li et al [36]. A similar proteolytic mechanism might be involved in the regulation of TRIF levels in poly(I:C) stimulated SAEC and might provide a mechanism to negatively regulate the cellular response to poly(I:C). However, currently we can not exclude that a poly(I:C) induced modification of TRIF might prevent the antibody detection of modified TRIF in our Western blot experiments. We found that TRIF degradation or modification was detectable after 3 h of poly(I:C) stimulation of SAEC and proceeded the degradation of IRAK-1, which is in agreement that IRAK-1 activation is down-stream of TRIF in TLR3 signaling.

In addition to the increased expression of TLR3, poly(I:C) has also a strong impact on the expression of other TLRs in SAEC. We could demonstrate that poly(I:C) strongly increased the expression of TLR2, whereas the expression of TLR5 and TLR6 was down-regulated by poly(I:C) stimulation. TLR1 expression was found to be strongly down-regulated after 6 h of poly(I:C) stimulation followed by an elevated mRNA and protein expression of TLR1 after 24 h of stimulation. The increased expression of TLR1 and TLR2 and the simultaneous down-regulation of TLR6, indicates that TLR3 activation favors a signaling through TLR1/TLR2 heterodimers rather than TLR2/TLR6 dimers. Furthermore, we could demonstrate that poly(I:C) stimulation results in an increased expression of IRAK-2. IRAK-2 has been shown to interact with TIRAP [37], which is involved in TLR2 and TLR4 signaling [38,39]. Although IRAK-2 posses no kinase activity, overexpression of IRAK-2 has been shown to promote NFκ-B activation [40]. Therefore, these poly(I:C) induced effects are likely to affect TLR2 and TLR4 mediated immune response in infected airways that have to be analyzed more carefully. Likewise, the down-regulation of TLR5 by poly(I:C) and the consequence for pulmonary infections with flagellated bacteria needs further investigations.

Our data demonstrate a pronounced regulation of the expression of TLRs and TLR signaling proteins in SAEC by type-1 and type-2 cytokines, which is important considering the impact of exogenous (pathogen associated) or endogenous TLR ligands on Th1 or Th2 driven pulmonary inflammations like COPD or asthma, respectively. Our data demonstrate that IFN-γ stimulation of SAEC results in the induction of an elevated IRAK-2, MyD88 and TRIF mRNA and protein expression and leads to the down-regulation of TLR1, TLR5 and TLR6 expression. There is a strong synergism of IFN-γ and TNF-α that results in a highly elevated expression of TLR2 and an increased expression of TLR4. Likewise, TNF-α alone or in combination with IFN-γ up-regulates TLR1 expression in SAEC. These data indicate that a type-1 cytokine milieu (i.e. TNF-α together with IFN-γ) promotes TLR2 and TLR4 signaling due to an up-regulation of the TLR1/TLR2 and TLR4 expression as well as due to an enhanced expression of MyD88, TRIF and IRAK-2. On the other side, type-1 cytokines are likely to inhibit TLR5 and TLR2/TLR6 signaling due to the strong down-regulation of TLR5 and TLR6 expression in SAEC. The observed up-regulation of IRAK-3, a negative regulator of TLR signaling [41], by type-1 cytokines might represent a self-limiting mechanism helping to control the inflammatory response of SAEC.

We also analyzed the influence of a Th2 cytokine milieu on the mRNA expression of TLR and their signaling or proteins. These data demonstrate that type-2 cytokines like IL-4 and IL-13 induce an elevated gene expression of TLR4 in SAEC and, together with TNF-α, up-regulate the gene expression of TLR1 and TLR2 in a synergistic manner. Therefore, this cytokine milieu is likely to promote signaling through TLR1/TLR2 heterodimers. The elevated expression of TLR4 and TLR1/TLR2 induced by type-2 cytokines is assumed to modulate the impact of bacterial infections on Th2 associated pulmonary diseases like asthma. In this regard, it is interesting to note that bacterial TLR2 and TLR4 ligands like lipopeptides or LPS have been shown to modulate the immune response in animal models of allergic asthma. Whereas the TLR1/TLR2 specific ligand Pam_3_CSK_4 _and high concentrations of the TLR4 ligand *E. coli *LPS have beneficial effects in asthma animal models [12-14], low-dose LPS and the TLR2 ligand peptidoglycan bias the immune response toward a Th2 phenotype and lead to aggravation of experimental allergic asthma [12,15].

## Conclusion

In summary, our data demonstrate that among the evaluated TLR ligands, poly(I:C), a synthetic analog of double-stranded viral RNA mediates the strongest proinflammatory effects in primary small airway epithelial cells (SAEC) with respect to cytokine and chemokine secretion and MMP release of the cells. These inflammatory features of poly(I:C) are thought to contribute to viral exacerbations of pulmonary inflammations including COPD and asthma. Furthermore, poly(I:C) modulates the gene expression of other TLRs in SAEC, what is likely to have a strong influence on the response of the lung epithelium to stimulation with additional TLR ligands during viral and bacterial infections. In addition, our data demonstrate a pronounced regulation of the expression of TLRs and TLR signaling proteins by a type-1 or type-2 cytokine milieu. The regulation of TLR expression in small airway epithelial cells by type-1 and type-2 cytokines is important considering the impact of exogenous (pathogen associated) or endogenous TLR ligands on Th1 or Th2 driven pulmonary inflammations like COPD or asthma, respectively.

## List of abbreviations

COPD: chronic obstructive pulmonary disease; dsRNA: double-stranded RNA; IRAK: Interleukin-1 receptor associated kinase; MALP-2: macrophage activating lipopeptide-2; MMP: matrix metalloproteinase; Pam_3_CSK_4_: synthetic tripalmitoylated lipopeptide Pam_3_CysSer(Lys)_4_; PGN: peptidoglycan; poly(I:C): polyinosine-polycytidylic acid; SAEC: small airway epithelial cell.

## Competing interests

The work was supported by Boehringer-Ingelheim Pharma GmbH & Co. KG, Germany.

## Authors' contributions

MR conceived of the experiments, carried out the experimental work of the study and drafted the manuscript. PS conceived of the experiments, participated in the design and direction of the study and revised the manuscript. AW and DM made substantial contribution to the data interpretation and helped to revise the manuscript. All authors read and approved the final manuscript.
